# Administration of Direct Oral Anticoagulant Immediately after Unfractionated Heparin Bolus for the Treatment of Intermediate–High-Risk Pulmonary Thromboembolism

**DOI:** 10.3400/avd.oa.20-00079

**Published:** 2020-12-25

**Authors:** Nobuhiro Hara, Keita Watanabe, Ryoichi Miyazaki, Tomofumi Nakamura, Tetsumin Lee, Yasutoshi Nagata, Toshihiro Nozato, Takamichi Miyamoto, Toru Obayashi, Takashi Ashikaga

**Affiliations:** 1 Department of Cardiology, Japanese Red Cross Musashino Hospital, Tokyo, Japan; 2 Department of Clinical Engineering, Gunma Paz College, Takasaki, Gunma, Japan

**Keywords:** anticoagulation, therapy, direct oral anticoagulant, intermediate–high-risk pulmonary embolism

## Abstract

**Objective**: This study aims to evaluate the efficacy and safety of direct oral anticoagulants (DOACs) after unfractionated heparin (UFH) bolus for the treatment of intermediate–high-risk pulmonary embolism.

**Materials and Methods**: On the basis of initial treatment, 81 patients were divided into two groups: DOAC after UFH bolus infusion group (group D; n=32) and conventional therapy group (group C; n=49). The frequency of recurrence of venous thromboembolism (VTE) and bleeding within 6 months were compared. In addition, hospitalization length and thrombus reduction rate in the pulmonary artery on computed tomography (CT) at the chronic phase were assessed.

**Results**: Recurrence of VTE was found in one (3.1%) and three patients (6.1%) (P=1.00) in groups D and C, respectively, whereas no bleeding events was found in group D and 8.2% of patients in group C (P=0.15). Group D showed shorter hospitalization (7.2±2.3 days) than group C (15.7±9.9 days; P<0.001). In the subset of patients with serial CT assessment (group D, n=20; group C, n=38), almost all thrombus of pulmonary artery were disappeared and the thrombus reduction rates were similar between the two groups (group D, 99.5%; group C, 97.1%; P=0.59).

**Conclusion**: DOAC administration immediately after UFH bolus treatment has the same efficacy and safety, whereas hospitalization days were significantly shorter than the conventional treatment group.

## Introduction

Pulmonary embolism (PE) is a fatal disease with high mortality in severe cases.^1,2)^ A high mortality rate of 7.7% has been reported for intermediate–high-risk PE.^3)^ According to 2019 ESC guidelines,^4)^ the classification of severity of PE and the risk of early (hospital or 30-day) death are as follows: (1) Hemodynamic instability is high risk. (2) Pulmonary Embolism Severity Index (PESI) class III–V or simplified PESI ≥I is intermediate high risk. Patients in the intermediate-risk group who display evidence of both right ventricular (RV) dysfunction (on echocardiography or computed tomography [CT]) and elevated cardiac biomarker levels in the circulation (particularly a positive cardiac troponin test) are classified into the intermediate–high-risk category. (3) Low risk is PESI of I–II or simplified PESI of 0 and no RV dysfunction. For acute-phase treatment of high-risk PE, it is recommended that anticoagulation with unfractionated heparin (UFH), including a weight-adjusted bolus injection, be initiated without delay and systemic thrombolytic therapy. Surgical pulmonary embolectomy is recommended in whom thrombolysis is contraindicated or has failed. Extracorporeal membrane oxygenation (ECMO) may be considered, in combination with surgical embolectomy or catheter-directed treatment, in patients with PE and refractory circulatory collapse or cardiac arrest. The recommended treatment of intermediate- or low-risk PE is anticoagulation. Rescue thrombolytic therapy is recommended for patients with hemodynamic deterioration on anticoagulation treatment. As an alternative to rescue thrombolytic therapy, surgical embolectomy or percutaneous catheter-directed treatment should be considered for patients with hemodynamic deterioration on anticoagulation treatment. Routine use of primary systemic thrombolysis is not recommended in patients with intermediate- or low-risk PE.

According to the Japanese Circulation Society guidelines, parenteral anticoagulants are the first choice for PE patients having normal blood pressure (BP) with RV dysfunction and positive cardiac biomarkers (intermediate–high-risk PE). These patients should also be monitored for signs of hemodynamic deterioration. If hemodynamic deterioration is observed, thrombolytic therapy can also be considered.

The efficacy and safety of direct oral anticoagulant (DOAC) for venous thromboembolism (VTE) have been reported.^5–8)^ On the other hand, conventional treatment (i.e., intravenous continuous heparin infusion and warfarin) is still an available option in real-world setting because of cheaper cost or contraindication of DOAC. Thus, in this study, we examined the therapeutic outcomes of DOAC immediately after UFH bolus for treatment of patients with intermediate–high-risk PE compared with conventional treatment using continuous intravenous UFH infusion treatment.

## Materials and Methods

### Patient population

We reviewed patient medical records and obtained data from Japanese Red Cross Musashino Hospital medical record. From January 2012 to December 2018, 83 of 413 PE patients were intermediate–high-risk PE. We excluded patients who were ineligible for anticoagulant therapy because of a history of hypersensitivity to anticoagulants, active hemorrhage (intracranial hemorrhage, retroperitoneal hemorrhage, hemorrhage from other important organs), acute bacterial endocarditis, creatinine clearance of <30 mL/min, or a diagnosis of chronic thromboembolic pulmonary hypertension. We also excluded one patient with loss of follow-up and one patient who had creatinine clearance of <30 mL/min. Finally, 81 patients with intermediate–high-risk PE were evaluated in this study, of which 32 were treated with DOAC immediately after UFH bolus (DOAC after UFH bolus group) and 49 with warfarin or DOAC after continuous intravenous heparin (conventional therapy group). We compared clinical characteristics, CT findings, and follow-up events between the two groups. The treatment method was divided according to the judgment of the attending physician. Rivaroxaban/apixaban could be used after October 2015 due to insurance adaptation issues; therefore, DOAC after UFH bolus group was cases after October 2015. All 81 patients were diagnosed by CT, and intermediate–high-risk PE were defined as follows: (1) normal BP, (2) right/left ventricle ratio ≥1.0 on CT, (3) brain natriuretic peptide ≥100 pg/mL or elevated troponin I (≥26 pg/mL), and (4) simplified PESI≧1.^4)^ Normal BP was defined as systolic BP ≥90 mmHg or systolic BP drop <40 mmHg.

**Figure 1:**
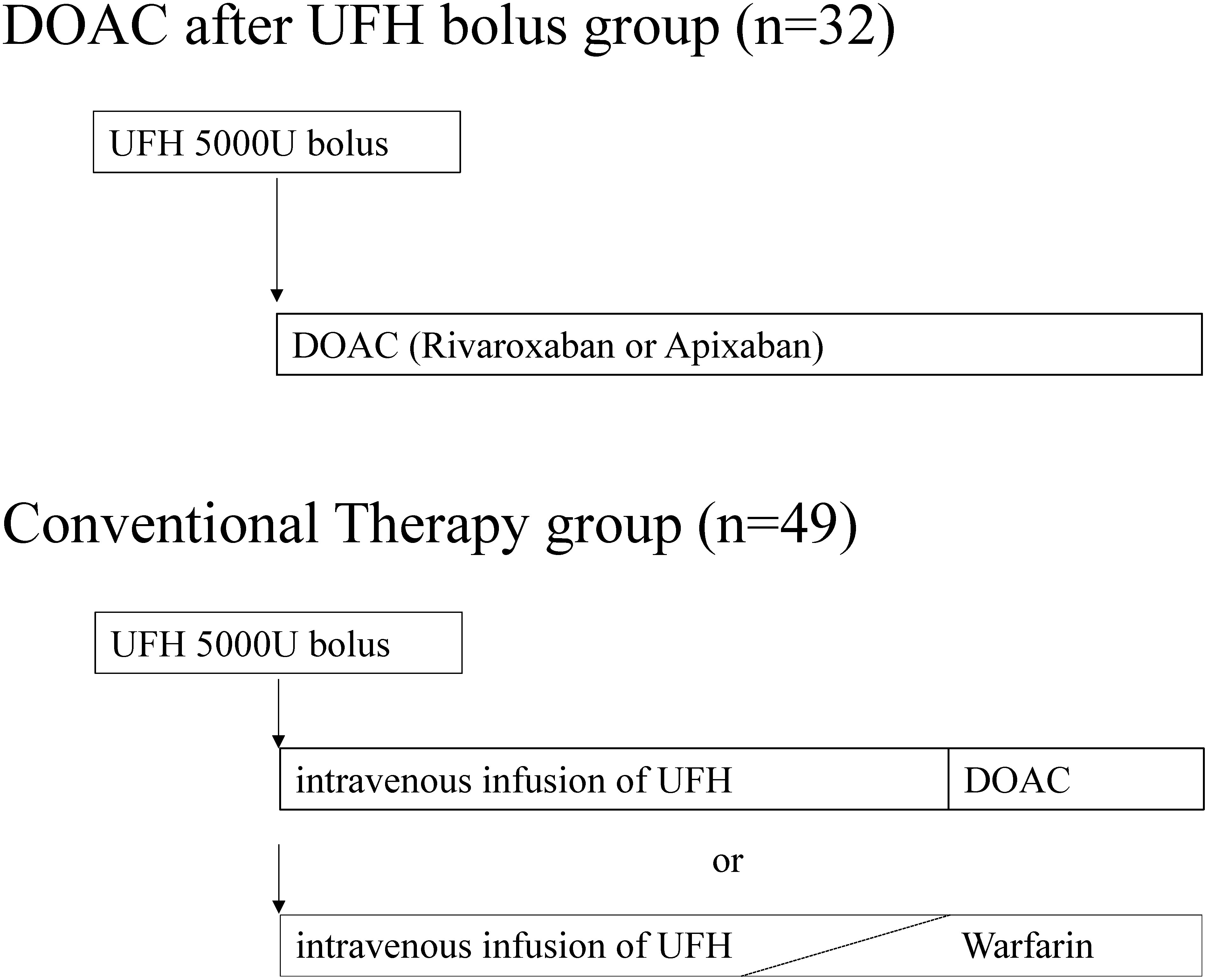
Fig. 1 Methods of treatment for intermediate–high-risk pulmonary thromboembolism.

In the DOAC after UFH bolus group, oral rivaroxaban or apixaban was administered within 30 min of intravenous injection of 5000 units of UFH. Rivaroxaban was administered twice per day at 15 mg for 21 days and then once per day at 15 mg. Apixaban was administered twice per day at 10 mg for 7 days and then twice per day at 5 mg. In the conventional therapy group, continuous intravenous heparin was administered after an intravenous injection of 5000 units of UFH. The dose was adjusted so that the activated partial thromboplastin time would be 1.5 times the control value, and the duration of administration was decided by the attending physician. The patients were then transitioned to warfarin (prothrombin time [PT]‐international normalized ratio [INR] 1.5–2.5) or DOAC. Warfarin was combined with UFH until PT-INR became ≥1.5 when UFH was subsequently terminated. DOAC was administered after terminating UFH (**Fig. 1**). Rivaroxaban was administered twice per day at 15 mg for 21 days and then once per day at 15 mg. Apixaban was administered twice per day at 10 mg for 7 days and then twice per day at 5 mg. Edoxaban was administered once per day at 60 mg or at 30 mg for patients who (1) weighed ≤60 kg; (2) were also taking quinidine sulfate hydrate, verapamil hydrochloride, erythromycin, or cyclosporine; or (3) had creatinine clearance of ≥30 mL/min and ≤50 mL/min. All patients underwent hospitalization and electrocardiogram monitoring and measurements for oxygen saturation with the attending physician’s discretion. Hospital discharge was decided by resolution of symptoms, improvement of right heart failure, and no adverse side effects from anticoagulant therapy.

Patients in the DOAC after UFH bolus group were administered oral DOAC (rivaroxaban or apixaban) immediately after UFH bolus. The conventional therapy group received oral warfarin or DOAC (rivaroxaban, apixaban, or edoxaban) after continuous intravenous UFH following a UFH bolus. Continuous intravenous UFH was administered for 6.5±2.5 days. UFH: unfractionated heparin; DOAC: direct oral anticoagulants

If the attending physician decided that treatments other than anticoagulant therapy were necessary, an inferior vena cava filter (IVCF) and tissue plasminogen activator (tPA) were used. For the prevention of fatal PE, IVCF was used in the case of presence of a floating thrombus in the lower extremity vein of the proximal region, especially in the iliac vein. Intravenous injection of tPA was used when respiratory or hemodynamics deteriorated.

Creatinine clearance was calculated using the Cockcroft–Gault formula. Heart disease was defined as a history of heart failure, ischemic heart disease, and arrhythmia. Brain disease was defined as a history of cerebral hemorrhage and cerebral infarction.

### Follow-up

Over a 6-month period, the primary outcomes, efficacy, and bleeding events were evaluated. The secondary outcomes were hospitalization length and thrombus reduction rate evaluated on contrast-enhanced CT at 3–6 months after the initiation of therapy. Efficacy was defined as recurrence of symptomatic venous thrombosis or VTE-related death. Recurrence of symptomatic venous thrombosis is defined as PE or deep vein thrombosis (DVT) or new thrombus found by contrast-enhanced CT or ultrasound images of veins of the lower extremities. VTE-related deaths were defined as deaths caused by PE or those that were unexplained but could not exclude VTE. Bleeding evaluation was based on the International Society on Thrombosis and Hemostasis criteria as clinically apparent acute hemorrhage, including at least one of the following: (1) fatal hemorrhage, (2) symptomatic hemorrhage at important sites or organs (intracranial, intrathecal, intraocular, retroperitoneal, intra-articular, pericardial, and intramuscular hemorrhage accompanied by compartment syndrome), and (3) decrease in hemoglobin by ≥2 g/dL and packed red blood cell transfusion of ≥2 units.

Thrombus reduction rate was calculated by measuring the volume of the thrombus in the pulmonary artery using an image analysis software (SYNAPSE VINCENT, Fujifilm Co., Ltd., Tokyo, Japan) at initial examination and during the chronic stage (3–6 months later). The thrombus reduction rate was calculated as follows:

(Initial thrombus volume−chronic-stage thrombus volume)/initial thrombus volume.

### Statistical analysis

Data normality was verified using the Kolmogorov–Smirnov test. Categorical data were expressed as absolute frequencies and percentages, and were compared using the χ^2^ or Fisher’s exact test, as appropriate. Continuous variables were expressed as means±standard deviation for normally distributed variables and as median values (25th–75th percentiles) for non-normally distributed variables, which were compared using Student’s t-test and the Mann–Whitney U test, respectively. Statistical analyses were performed using the R statistical package version 3.1.0 (The R Foundation for Statistical Computing, Vienna, Austria; http://www.r-project.org/). A two-sided P value of <0.05 was considered statistically significant.

### Ethical statement

This study conformed to the ethical principles of the Declaration of Helsinki. The requirement for informed consent was waived, because all data were anonymously cataloged. The institutional review board of the Japanese Red Cross Musashino Hospital approved the study protocol (protocol number, 1035; date of approval by the ethics committee, July 18, 2019). The information disclosure document associated with this study is available on the hospital’s website. Patients were notified about their participation in the study and were informed that they were free to opt out of the study at any time.

## Results

Demographic and clinical data of the subjects are shown (**Table 1**). No significant differences were observed in age, sex, weight, VTE risk factors, right/left ventricle ratio, medical history, or blood test. tPA was used in two patients (4.1%) in the conventional therapy group. Symptoms improved after intravenous injection of tPA. IVCF was used in a significantly higher proportion (40.8%) of patients in the conventional therapy group than in the DOAC after UFH bolus group (3.1%) (P<0.001). IVCF was used only during the acute phase and was discontinued in all patients who had no complications. In this study, no surgical embolectomy or percutaneous catheter-directed treatment was performed. In the conventional therapy group, continuous intravenous UFH was administered for 6.5±2.5 days, and the time in therapeutic range (TTR) was 65.3%. With warfarin, the TTR was 82.0%. In all cases, symptoms were improved with acute-phase treatment and discharged with anticoagulant therapy.

**Table 1:** Table 1 Baseline characteristics of study participants

	DOAC after UFH bolus group n=32	Conventional therapy n=49	P value
Age, mean (SD)	68.1 (12.3)	69.4 (15.7)	0.703
Male (%)	24 (75.0)	30 (61.2)	0.234
Body weight, mean (SD)	60.6 (12.1)	57.5 (13.3)	0.288
DVT (%)	23 (71.9)	40 (83.3)	0.269
Proximal DVT (%)	8 (27.6)	25 (52.1)	0.056
VTE risk factor			
Transient risk (%)	13 (40.6)	18 (36.7)	0.816
Unprovoked (%)	14 (43.8)	17 (34.7)	0.486
Active cancer (%)	5 (15.6)	14 (28.6)	0.283
RV/LV ratio (SD)	1.4 (0.2)	1.4 (0.2)	0.894
Troponin I, pg/mL	60.0 (35–260)	69.5 (40–141)	0.336
BNP, pg/mL	160 (72–400)	184 (55–499)	0.889
Hypertension (%)	16 (50.0)	17 (34.7)	0.247
Diabetes mellitus (%)	2 (6.2)	5 (10.2)	0.698
Dyslipidemia (%)	9 (28.1)	11 (22.4)	0.605
Heart disease (%)	2 (6.2)	11 (22.4)	0.066
Brain disease (%)	1 (3.1)	4 (8.2)	0.643
Antiplatelet (%)	1 (3.1)	3 (6.1)	1
Smoking (%)	5 (15.6)	10 (20.4)	0.771
CCR, mean (SD), mL/min	68.3 (22.4)	75.4 (31.9)	0.284
D dimer, mean (SD), µg/mL	9.5 (4.7)	12.6 (17.5)	0.326
WBC, mean (SD), /µL	7,920 (2546)	8,547 (2839)	0.316
RBC, mean (SD), ×10^4^/µL	428 (62)	410 (70)	0.269
Hb, mean (SD), g/dL	12.9 (2.4)	12.4 (2.7)	0.39
Ht, mean (SD), %	38.1 (6.0)	36.7 (6.8)	0.326
Plt, mean (SD), ×10^4^/µL	24.2 (13.7)	19.9 (8.3)	0.083
Additional treatment			
IVCF (%)	1 (3.1)	20 (40.8)	<0.001
tPA (%)	0 (0.0)	2 (4.1)	0.516
Oral anticoagulants			
Rivaroxaban (%)	13 (40.6)	6 (12.2)	
Apixaban (%)	19 (59.4)	4 (8.2)	
Edoxaban (%)		6 (12.2)	
Warfarin (%)		33 (67.3)	

The data are expressed as numbers (%), means and standard deviations, or medians and interquartile ranges. DOAC: direct oral anticoagulant; UFH: unfractionated heparin; SD: standard deviation; DVT: deep vein thrombosis; VTE: venous thromboembolism; RV: right ventricle; LV: left ventricle; BNP: brain natriuretic peptide; CCR: creatinine clearance; WBC: white blood cell count; RBC: red blood cell count; Hb: hemoglobin; Ht: hematocrit; Plt: platelet count; IVCF: inferior vena cava filter; tPA: tissue plasminogen activator

Regarding efficacy, recurrence was only seen in one patient (3.1%) in the DOAC after UFH bolus group and in three (6.1%) in the conventional therapy group (P=1.00) (**Table 2**). In the DOAC after UFH bolus group, it was a case of PE that recurred in the chronic stage after the patient discontinued the medication themselves. In the conventional therapy group, three cases of PE recurred in the chronic stage, two of which were PE recurrences after patients discontinued the medication themselves. In one case, symptoms improved with acute-phase treatment, but PE recurrence occurred in the chronic stage despite taking warfarin. Bleeding was not seen in any patients from the DOAC after UFH bolus group, while four patients (8.2%) in the conventional therapy group had signs of hemorrhage (P=0.149) (**Table 2**). In all four patients, the bleeding occurred while taking oral anticoagulants in the chronic stage: three with warfarin and one with DOAC.

**Table 2:** Table 2 Primary outcomes

	DOAC after UFH bolus n=32	Conventional therapy n=49	P value
Efficacy (%)	1 (3.1)	3 (6.1)	1
Bleeding (%)	0 (0.0)	4 (8.2)	0.149

Efficacy was defined as recurrence of symptomatic venous thrombosis or VTE-related death. VTE-related deaths were defined as deaths diagnosed as caused by PE or those that are unexplained but in which VTE cannot be excluded as the cause. Hemorrhage was defined as clinically apparent acute hemorrhage, including at least one of the following: (1) fatal hemorrhage, (2) symptomatic hemorrhage at important sites or organs (intracranial, intrathecal, intraocular, retroperitoneal, intra-articular, pericardial, intramuscular hemorrhage accompanied by compartment syndrome), and (3) decrease in hemoglobin level by ≥2 g/dL and packed red blood cell transfusion of ≥2 units. The differences were not significant (Fisher’s exact test). UFH: unfractionated heparin; DOACs: direct oral anticoagulants; VTE: venous thromboembolism; PE: pulmonary embolism

**Figure 2:**
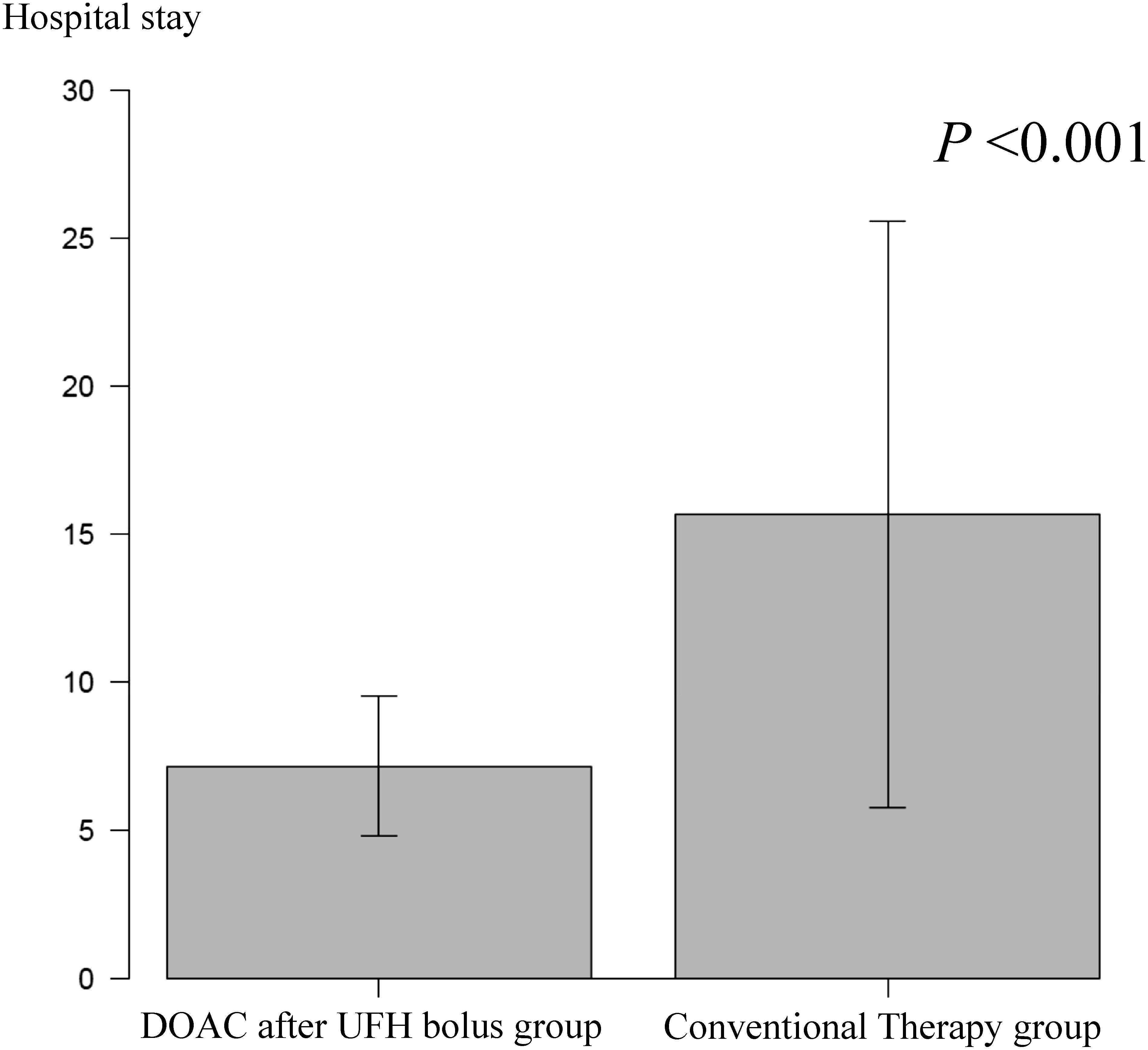
Fig. 2 Length of hospitalization for the two groups.

Hospital stay was significantly shorter in the DOAC after UFH bolus group (7.2±2.3 days) than in the conventional therapy group (15.7±9.9 days; P<0.001) (**Fig. 2**). Hospital stay was also significantly shorter in the DOAC after UFH bolus group than in the conventional therapy group who used DOAC (16 patients), at 13.1±6.2 days (P<0.001).

The hospitalization days of the DOAC after UFH bolus group was 7.2±2.3 days, which is significantly shorter than the 15.7±9.9 days in the conventional therapy group (P<0.001) (student t-test). UFH: unfractionated heparin; DOAC: direct oral anticoagulants

**Figure 3:**
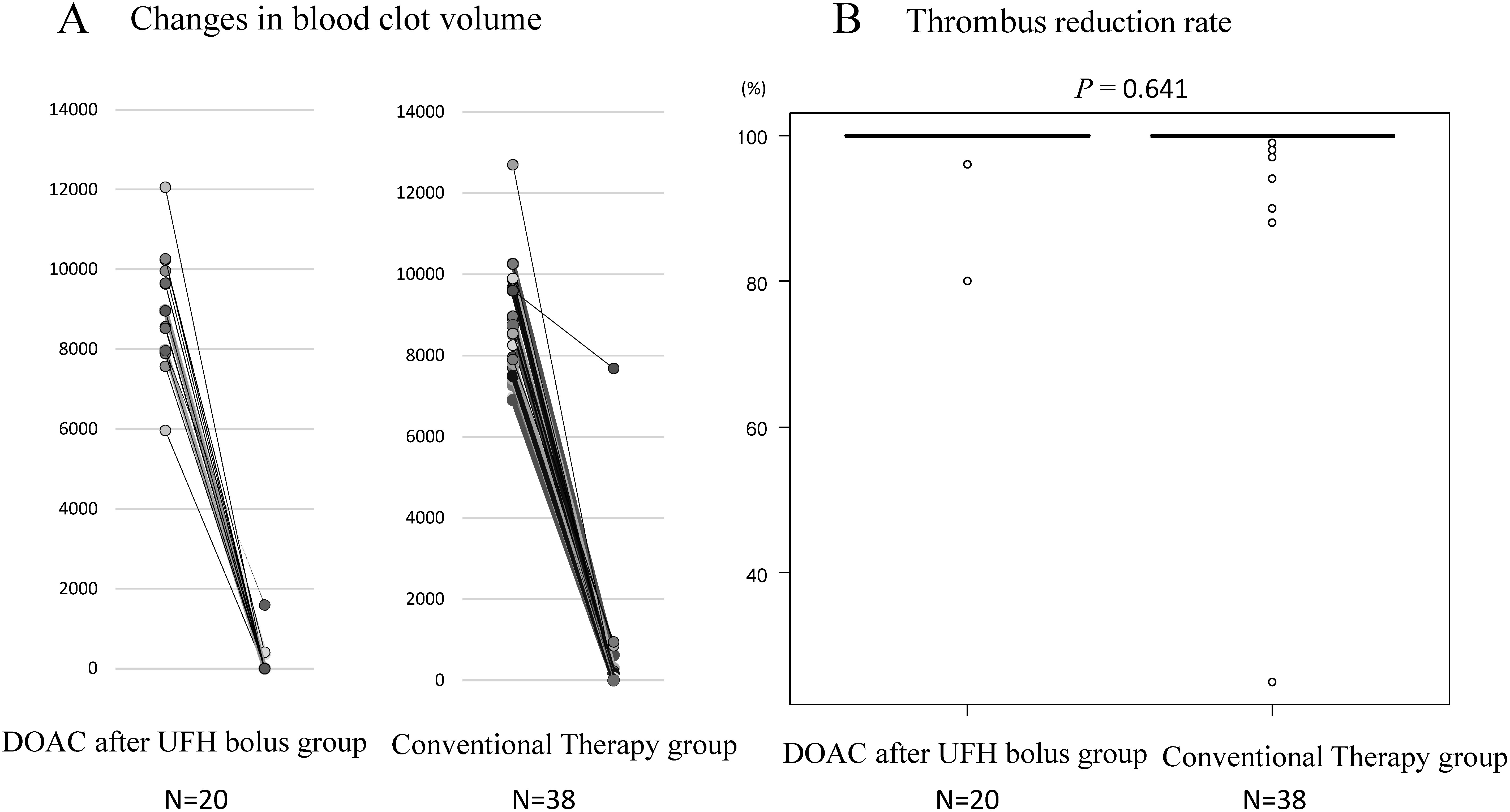
Fig. 3 Thrombus reduction rate after anticoagulation therapy.

CT was performed 3–6 months after the initiation of treatment in 20 patients (62.5%) from the DOAC after UFH bolus group and 38 patients (77.6%) from the conventional therapy group. The clot volume in DOAC after UFH bolus group was 8964 mm^3^ (8963 [interquartile range (IQR), 7966–9963]), while the volume for the conventional therapy group was 8694 mm^3^ (8544 [IQR, 7924–9600]). At the chronic phase, clot volume was 118 (0 [IQR, 0–0]) and 285 mm^3^ (0 [IQR, 0–0]), respectively (**Fig. 3A**). The thrombus reduction rates were 98.6% (100 [IQR, 100–100]) and 97.1% (100 [IQR, 100–100]), respectively (P=0.641) (**Fig. 3B**). Excluding one patient who stopped anticoagulant therapy themselves in DOAC after UFH bolus group, the thrombus reduction rates were 99.6%±1.3%. In four cases of recurrence, the symptoms improved in the acute phase and CT showed reduced thrombus, but thrombosis increased due to chronic stage recurrence. In cases where the blood clot is reduced with treatment, there were no symptoms.

CT was performed in the chronic stage on 20 patients (62.5%) in the UFH bolus group and 38 patients (77.6%) in the conventional therapy group. The clot volume in DOAC after UFH bolus group was 8964 mm^3^ (8963 [IQR, 7966–9963]), while the volume for the conventional therapy group was 8694 mm^3^ (8544 [IQR, 7924–9600]). At the chronic phase, clot volume was 118 (0 [IQR, 0–0]) and 285 mm^3^ (0 [IQR, 0–0]), respectively (**A**). The thrombus reduction rates were 98.6% (100 [IQR, 100–100]) and 97.1% (100 [IQR, 100–100]), respectively (P=0.641) (**B**) (Mann–Whitney U test). UFH: unfractionated heparin; DOAC: direct oral anticoagulants; CT: computed tomography; IQR: interquartile range

## Discussion

In this study, we administered a single intravenous UFH injection followed by DOAC in intermediate–high-risk PE patients with large thrombus volumes. These patients exhibited comparable efficacy and safety outcomes to patients who underwent conventional therapy. Notably, they had shorter hospital stays and higher thrombus reduction rates in the pulmonary artery 3–6 months posttreatment.

### Advantage of DOAC after UFH bolus

The efficacy and safety of DOAC for VTE have been reported,^5–8)^ and its use in clinical settings has been approved. DOAC can be used in a single-drug approach without heparin.^9)^ The effectiveness of DOAC using a single-drug approach has been demonstrated in the treatment of advanced PE.^7)^

The advantages of this method are that anticoagulation therapy can be administered rapidly without the need for adjustment. VTE has been reported to have a high recurrence rate if the initial treatment is poor.^10)^ Heparin therapy for VTE is reported to be below the therapeutic target range in 37.6% of cases.^11)^ In the present study as well, UFH did not reach TTR in 34.7% patients in the conventional therapy group. In Japan, health insurance does not cover the use of low-molecular-weight heparin that does not require dose adjustment, which is the anticoagulation therapy for PE. UFH has conventionally been used for the initial treatment; however, dose adjustment may be difficult in continuous intravenous UFH.

Early anticoagulant therapy has been reported to reduce mortality.^12)^ During initial therapy with oral DOAC, it takes several hours after administration for blood levels to rise sufficiently.^13)^ Administering UFH after contrast-enhanced CT is easy, and blood levels of UFH peak immediately upon intravenous administration^14)^ with a half-life of 45–60 min. The anticoagulant effect of UFH is rapid, and oral administration of DOAC immediately after a UFH bolus is effective for achieving a sustained anticoagulant effect. DOAC immediately after a UFH bolus is considered particularly important for intermediate–high-risk PE patients in severe condition. Without enhanced initial therapy, the recurrence rate is reported to be higher^15)^; therefore, rivaroxaban or apixaban was used in the DOAC after UFH bolus group. Thrombus volume improvement on DOAC has been reported in VTE.^16)^ Similarly, a high rate of pulmonary thrombus reduction on CT after 3–6 months was observed in the present study in the both groups.

Caution is needed with conditions, such as intestinal edema, as the drug may not be absorbed into the body with oral administration. Regarding hypercoagulability cases, such as COVID-19^17)^ or active cancer, further trials are needed to determine if fixed-dose anticoagulants are effective.

### Treatments other than anticoagulant therapy for intermediate–high-risk PE

It has been reported that even mild cases of PE with stable hemodynamics can have extensive residual DVT, and when a DVT advances centrally owing to insufficient treatment, recurrence of embolization becomes serious.^18)^ Using filters has been reported to be useful in reducing the mortality in acute PE.^2)^ As the recurrence of embolization of a residual thrombus may be fatal in intermediate–high-risk PE patients, using IVCF should be appropriately considered in accordance with the guidelines. The rate of IVCF use has been declining since its peak in 2010.^19)^ In the DOAC after UFH bolus group, PE patients could only receive rivaroxaban starting October 2015. Therefore, the difference in IVCF rates was likely due to a tendency toward fewer cases of proximal DVT in addition to the trend of a lower frequency of IVCF use. While tPA was only used in two patients in the conventional therapy group, this can be considered if the improvement is inadequate with anticoagulant therapy alone or if a new thromboembolism becomes severe.^20)^

### Hospitalization days for intermediate–high-risk PE

Hospitalization was shorter in patients undergoing treatment with rivaroxaban than patients treated with enoxaparin/warfarin.^21,22)^ The reason for early discharge is that dose adjustment is not required for rivaroxaban/apixaban. In the present study, hospitalization was shorter even in patients who transitioned to oral DOAC after continuous intravenous UFH. Another reason why hospitalization was shorter is because the follow-up period after switching from the infusion to oral DOAC was unnecessary.

### Limitations

First, this was a retrospective study conducted in a single institution with small sample size. Therefore, selection bias was inevitable. A multicenter randomized study with larger sample size would be required to generalize our results. Second, some patients did not undergo CT assessment in the chronic stage and the time periods of CT assessment between index of PE and follow-up stage were different in each patient. Lastly, we did not evaluate the prevalence of DVT in the lower limbs.

## Conclusion

DOAC administration immediately after UFH bolus for the treatment of intermediate–high-risk PE has the same efficacy and safety, and hospitalization days were significantly shorter than the conventional treatment group in real-world setting.
